# Supplementary material for: Deep learning for robust and flexible tracking in behavioral studies for C. elegans
Source: PLoS Comput Biol. 2022 Apr 8;18(4):e1009942. doi: 10.1371/journal.pcbi.1009942 (PMC9020731; doi:10.1371/journal.pcbi.1009942)
Supplement: S1 Fig — A. Detection of animals throughout development using the trained WoP Faster R-CNN model or a common tool that uses traditional segmentation (Tierpsy Tracker). Tierpsy Tracker parameters were manually tuned to detect the animal in the example image in A, and not re-tuned for analyzing the same animal at later timepoints in B-E. Successful segmentation of the worm by Tierpsy Tracker is denoted by a white arrow, with non-worm segmentations marked by red arrows (middle column). Worm detections using the WoP Faster R-CNN model are bounded by a red box (left column). All worm detections shown reached a confidence threshold of 0.99. The animal in A is detected by both the WoP model and Tierpsy Tracker, but other non-worm objects are identified based on the optimized Tierpsy Tracker segmentation. B. The same animal as in A at a later timepoint. The animal is identified using the WoP model, but not identified by Tierpsy Tracker. C. As contrast improves, the same animal is detected by both Tierpsy Tracker and the WoP model, but the segmentation parameters as optimized for small, low-contrast animals also pick up non-worm objects. D. Once the animal becomes a gravid adult, the animal is identified by both Tierpsy Tracker and the WoP model, but eggs and tracks in the bacterial lawn increase the number of non-worm segmentations by Tierpsy Tracker. E. Illumination changes increases the number of non-worm segmentations by Tierpsy Tracker, while the WoP model is still able to identify the animal and no other non-worm objects. (PDF) [file pcbi.1009942.s001.pdf]

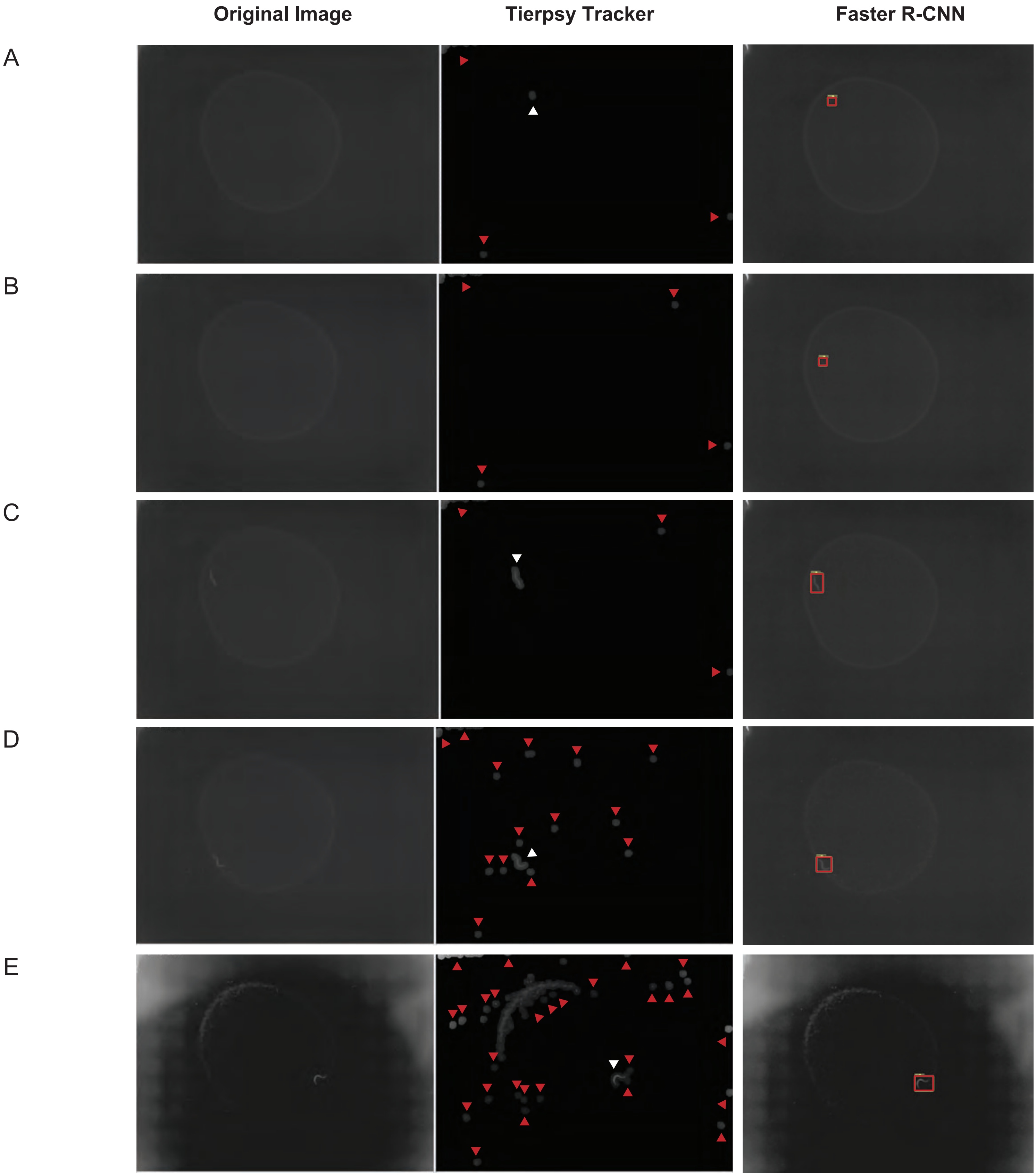

Original Image
Tierpsy Tracker
Faster R-CNN
A
B
C
D
E

Supplemental Figure 1. Limitations of traditional image processing techniques in developmental monitoring

A. Detection of animals throughout development using the trained WoP Faster R-CNN model or a common tool that uses traditional segmentation (Tierpsy Tracker). Tierpsy Tracker parameters were manually tuned to detect the animal in the example image in A, and not re-tuned for analyzing the same animal at later timepoints in B-E. Successful segmentation of the worm by Tierpsy Tracker is denoted by a white arrow, with non-worm segmentations marked by red arrows (*middle column*). Worm detections using the WoP Faster R-CNN model are bounded by a red box (*left column*). All worm detections shown reached a confidence threshold of 0.99. The animal in A is detected by both the WoP model and Tierpsy Tracker, but other non-worm objects are identified based on the optimized Tierpsy Tracker segmentation.
B. The same animal as in A at a later timepoint. The animal is identified using the WoP model, but not identified by Tierpsy Tracker.
C. As contrast improves, the same animal is detected by both Tierpsy Tracker and the WoP model, but the segmentation parameters as optimized for small, low-contrast animals also pick up non-worm objects.
D. Once the animal becomes a gravid adult, the animal is identified by both Tierpsy Tracker and the WoP model, but eggs and tracks in the bacterial lawn increase the number of non-worm segmentations by Tierpsy Tracker.
E. Illumination changes increases the number of non-worm segmentations by Tierpsy Tracker, while the WoP model is still able to identify the animal and no other non-worm objects.
