# Supplementary material for: Deep learning for robust and flexible tracking in behavioral studies for C. elegans
Source: PLoS Comput Biol. 2022 Apr 8;18(4):e1009942. doi: 10.1371/journal.pcbi.1009942 (PMC9020731; doi:10.1371/journal.pcbi.1009942)
Supplement: S2 Fig — A. Detection of a young worm using traditional techniques (Tierpsy Tracker) or the trained WiCh Faster R-CNN model (top row) Successful detection of a worm via Tierpsy Tracker. Parameters for traditional techniques were manually tuned for this specific video. (bottom rows) Detection of worms (under the same age and environmental condition) using the same parameters as before. Detection errors are highlighted by red arrows. Red boxes show detection location via WiCh Faster R-CNN model. B. Detection of an old, slow moving worm using traditional techniques (Tierpsy Tracker) or the trained WiCh Faster R-CNN model (top row) Successful detection of a worm. Parameters for traditional techniques were manually tuned for this specific video. (bottom rows) Detection of worms (under the same age and environmental condition) using the same parameters as before. Errors and misidentification are highlighted by red arrows. Red boxes show detection location via WiCh Faster R-CNN model. (PDF) [file pcbi.1009942.s002.pdf]

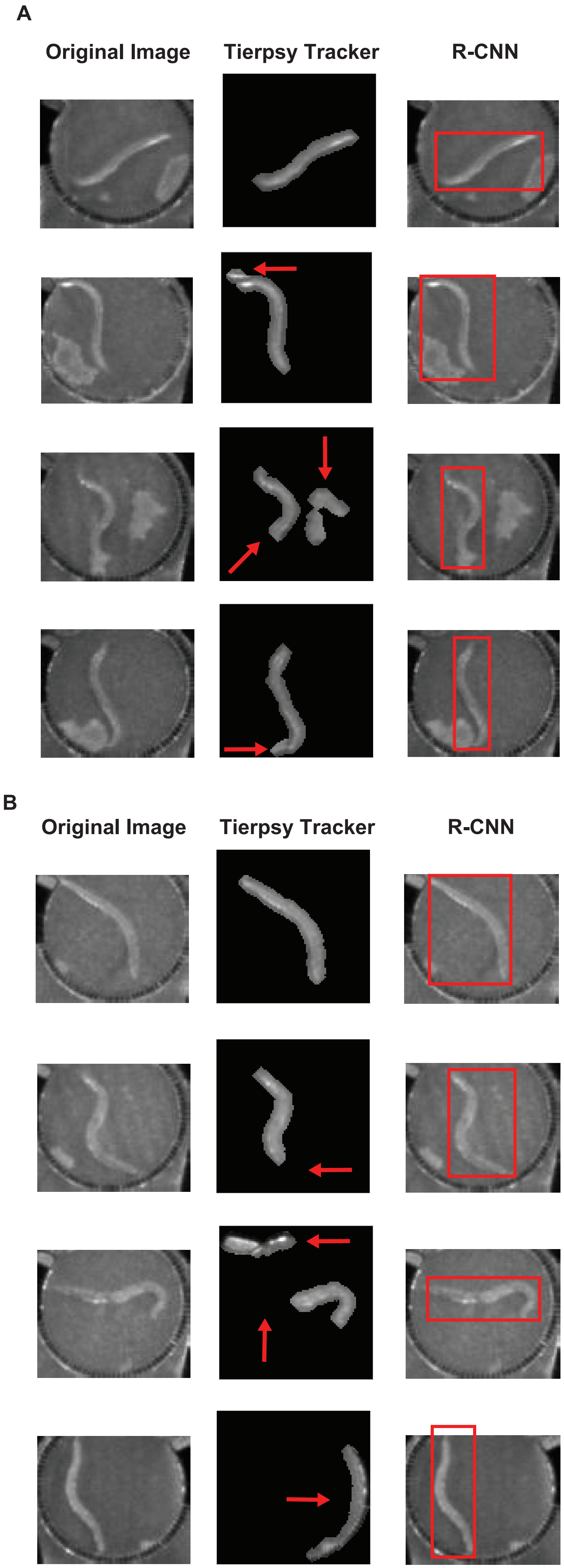
A
Original Image
Tierpsy Tracker
R-CNN
B
Original Image
Tierpsy Tracker
R-CNN

Figure S2. Limitations of traditional image processing techniques in aging populations

A. Detection of a young worm using traditional techniques (Tierpsy Tracker) or the trained Faster R-CNN model (top row) Successful detection of a worm via Tierpsy Tracker. Parameters for traditional techniques were manually tuned for this specific video. (bottom rows) Detection of worms (under the same age and environmental condition) using the same parameters as before. Detection errors are highlighted by red arrows. Red boxes show detection location via Faster R-CNN model.

B. Detection of an old, slow moving worm using traditional techniques (Tierpsy Tracker) or the trained Faster R-CNN model (top row) Successful detection of a worm. Parameters for traditional techniques were manually tuned for this specific video. (bottom rows) Detection of worms (under the same age and environmental condition) using the same parameters as before. Errors and misidentification are highlighted by red arrows. Red boxes show detection location via Faster R-CNN model.
