# Supplementary material for: Deep learning for robust and flexible tracking in behavioral studies for C. elegans
Source: PLoS Comput Biol. 2022 Apr 8;18(4):e1009942. doi: 10.1371/journal.pcbi.1009942 (PMC9020731; doi:10.1371/journal.pcbi.1009942)
Supplement: S3 Fig — A. Precision-recall curves for worm detection in the WoP model with confidence threshold of 0.5. Precision recall curve for all worms (left), L2-L3 stage animals (middle), and adult animals (right). B. Precision-recall curve for the worm detection (left) and egg detection (right) in the egg-finder model with confidence threshold of 0.01. The intersection over union used to determine true positive detections for eggs was 0.3, compared to 0.5 for worms. C. Precision-recall curve for the overall worm detection (left) and egg detection (right) in the WiCh model with confidence threshold of 0.5 (top row). Precision-recall curves for the worm at varying stages in the lifespan with confidence threshold of 0.5 (middle row), and across different food levels/contrasts with confidence threshold of 0.5 (bottom row). (PDF) [file pcbi.1009942.s003.pdf]

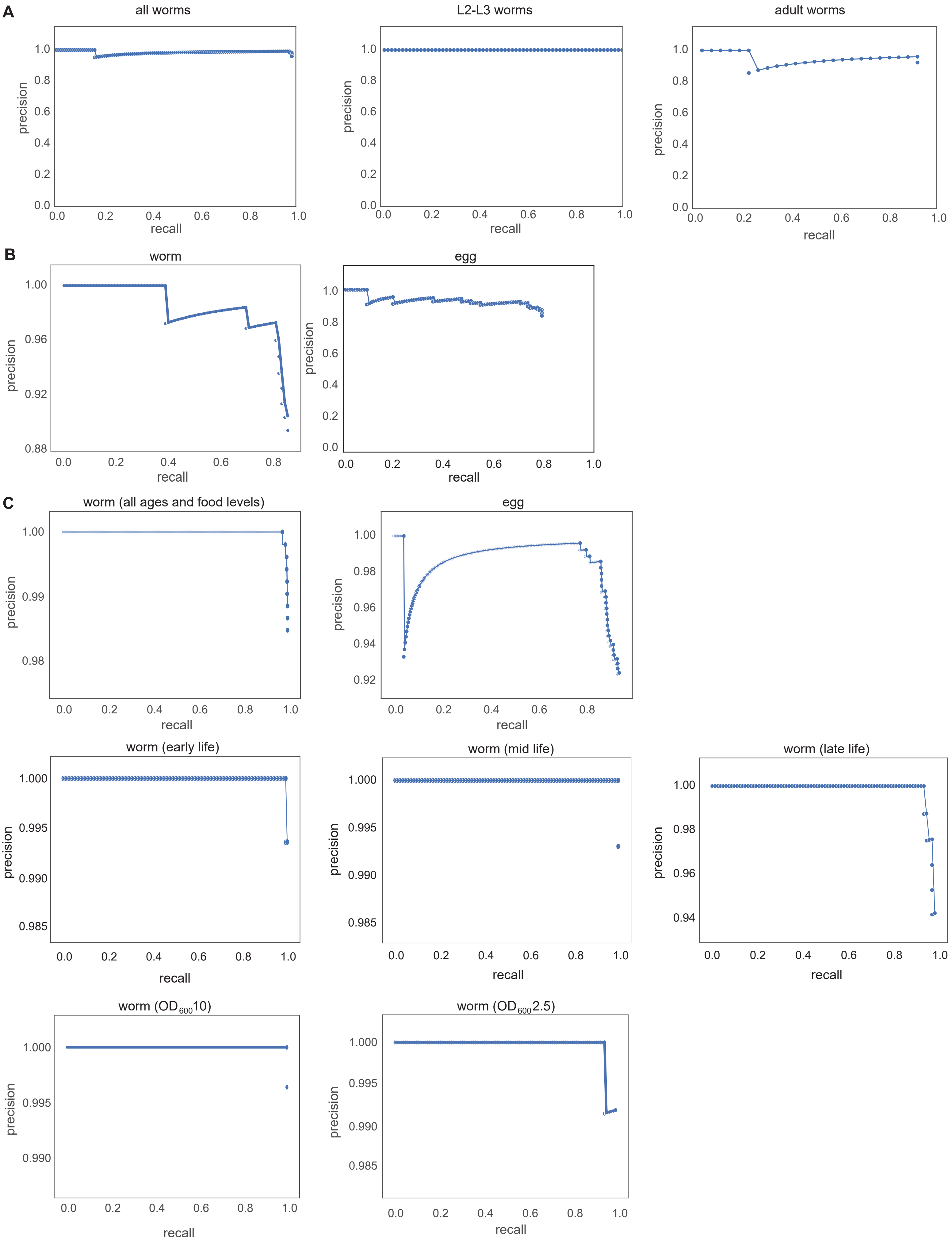
A
all worms
L2-L3 worms
adult worms
precision
recall
0.0
0.2
0.4
0.6
0.8
1.0
B
worm
egg
1.00
0.96
0.92
0.88
C
worm (all ages and food levels)
egg
0.99
0.98
0.94
0.92
worm (early life)
worm (mid life)
worm (late life)
1.000
0.995
0.990
0.985
worm (OD_{600}10)
worm (OD_{600}2.5)

Supplemental Figure 3. Precision-recall curves for the detection models

A. Precision-recall curves for worm detection in the WoP model with confidence threshold of 0.5. Precision recall curve for all worms (left), L2-L3 stage animals (middle), and adult animals (right).
B. Precision-recall curve for the worm detection (left) and egg detection (right) in the egg-finder model with confidence threshold of 0.01. The intersection over union used to determine true positive detections for eggs was 0.3, compared to 0.5 for worms.
C. Precision-recall curve for the overall worm detection (left) and egg detection (right) in the WiCh model with confidence threshold of 0.5 (top row). Precision-recall curves for the worm at varying stages in the lifespan with confidence threshold of 0.5 (middle row), and across different food levels/contrasts with confidence threshold of 0.5 (bottom row).
