# Supplementary material for: Deep learning for robust and flexible tracking in behavioral studies for C. elegans
Source: PLoS Comput Biol. 2022 Apr 8;18(4):e1009942. doi: 10.1371/journal.pcbi.1009942 (PMC9020731; doi:10.1371/journal.pcbi.1009942)
Supplement: S4 Fig — A. Comparison of X centroid coordinates from Stern et al. (2017) [13] to Faster R-CNN WoP model detections of the same data. Gap in Faster R-CNN detections at approximately 9 minutes occurs when animal collides with edge of circular arena. B. Comparison of Y centroid coordinates from Stern et al. (2017) [13] to Faster R-CNN WoP model detections of the same data. Gap in Faster R-CNN detections at approximately 9 minutes occurs when animal collides with edge of circular arena. (PDF) [file pcbi.1009942.s004.pdf]

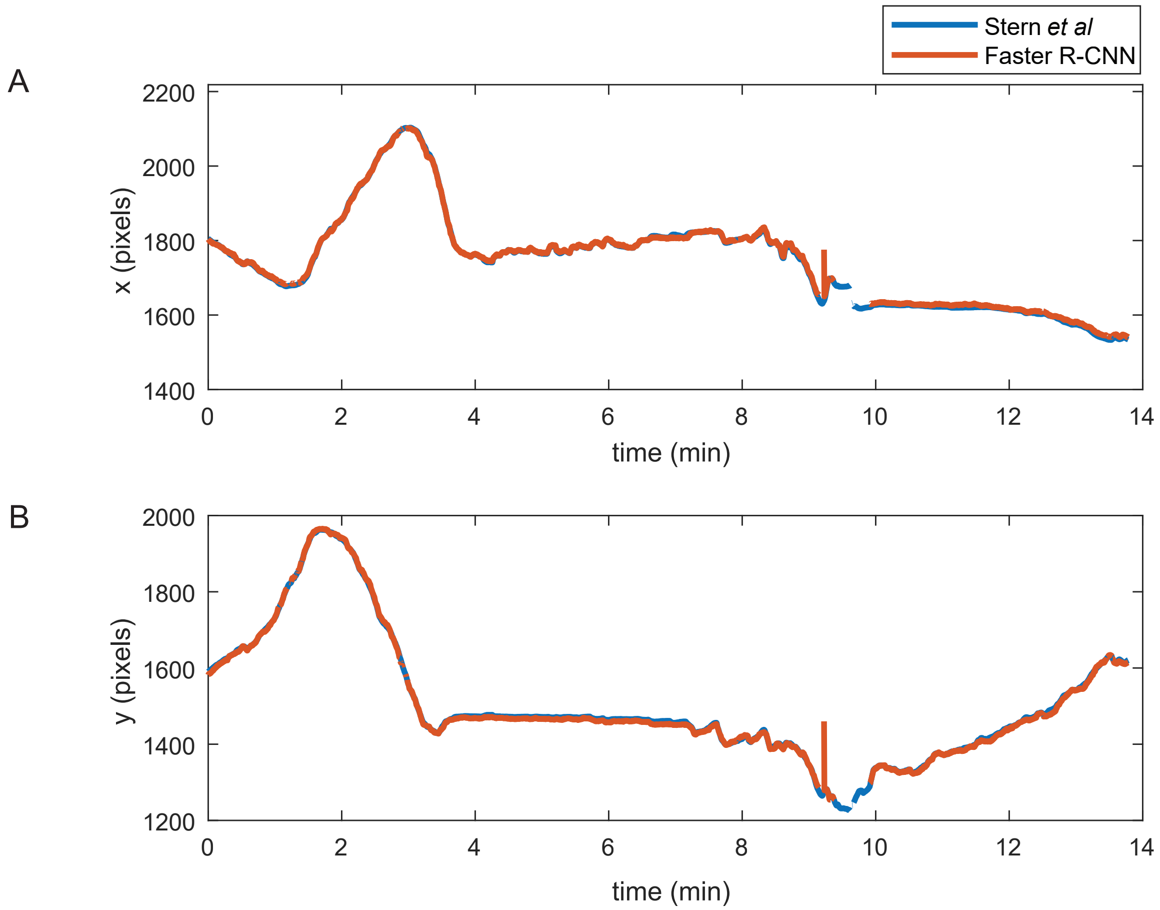

Supplemental Figure 4. Accurate centroid tracking in other datasets

A. Comparison of X centroid coordinates from Stern *et al.* to Faster R-CNN WoP model detections of the same data.

B. Comparison of Y centroid coordinates from Stern *et al.* to Faster R-CNN WoP model detections of the same data.
