# Supplementary material for: Deep learning for robust and flexible tracking in behavioral studies for C. elegans
Source: PLoS Comput Biol. 2022 Apr 8;18(4):e1009942. doi: 10.1371/journal.pcbi.1009942 (PMC9020731; doi:10.1371/journal.pcbi.1009942)
Supplement: S5 Fig — A. Comparison of binned angular velocity over time from animal and timepoints of animal in S4 Fig (data from Stern et al. (2017) [13]) and Faster R-CNN WoP detections of the same data. Data was binned by first smoothing angular velocities using a 10s moving average window (as in Stern et al. (2017) [13]), then thresholding the data into low and high angular velocities. The angular velocity values represent the average angular velocity of the low and high angular velocity data for each data set independently. B. Linear velocity of both datasets vs. time for animal and timepoints shown in S4 Fig. Linear velocity was calculated in the same way from both datasets, then smoothed with a moving average window of 10s, and finally by removing outliers. C. Scatterplot comparing Faster R-CNN WoP centroid velocities for animal and timepoints in S4 Fig to Stern et al. (2017) [13] ground truth velocities. Some structure is apparent because consecutive timepoints are likely to have correlated velocity, and in the case of the Faster R-CNN detection, correlated errors. (PDF) [file pcbi.1009942.s005.pdf]

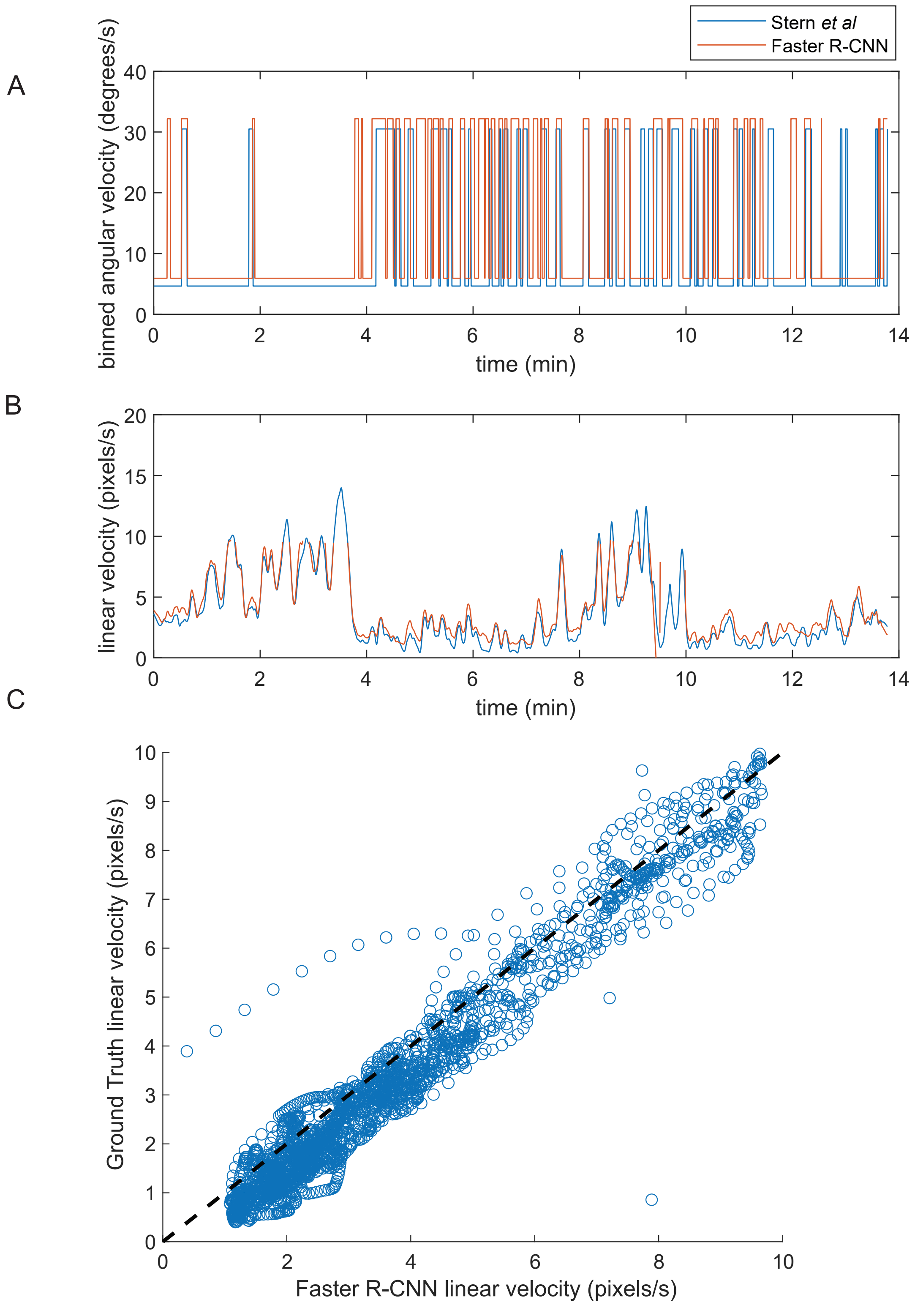

A
Stern et al
Faster R-CNN
binned angular velocity (degrees/s)
0
10
20
30
40
0
2
4
6
8
10
12
14
time (min)
B
linear velocity (pixels/s)
0
5
10
15
20
0
2
4
6
8
10
12
14
time (min)
C
Ground Truth linear velocity (pixels/s)
0
1
2
3
4
5
6
7
8
9
10
0
2
4
6
8
10
Faster R-CNN linear velocity (pixels/s)

Supplemental Figure 5. Accurate linear and angular velocity analysis with other datasets

A. Comparison of binned angular velocity over time from Stern *et al.* and Faster R-CNN WoP detections of the same data. Data was binned by first smoothing angular velocities using a 10s moving average window (as in Stern *et al*.), then thresholding the data into low and high angular velocities. The angular velocity values represent the average angular velocity of the low and high angular velocity data for each data set independently.
B. Linear velocity of both datasets vs. time. Linear velocity was calculated in the same way from both datasets, then smoothed with a moving average window of 10s, and finally by removing outliers.
C. Scatterplot comparing Faster R-CNN WoP centroid velocities to Stern *et al.* ground truth velocities.
