# Supplementary material for: Deep learning for robust and flexible tracking in behavioral studies for C. elegans
Source: PLoS Comput Biol. 2022 Apr 8;18(4):e1009942. doi: 10.1371/journal.pcbi.1009942 (PMC9020731; doi:10.1371/journal.pcbi.1009942)
Supplement: S6 Fig — A. Change of bounding box size over time using WoP Faster R-CNN for animal depicted in 4A. Smoothed using a moving window average over 10 time points (10 minutes). Where no animal is detected, line is not connected. B. Density histogram of distances between box centroid as detected by WoP Faster R-CNN model and centroid of annotated worm shape for animal in 4A (n = 30 time points). C. Density histogram of difference between movement calculated from annotations and movement calculated from WoP Faster R-CNN model for animal in 4A (n = 25 time points). D. Centroid movement as calculated by the WoP Faster R-CNN model vs. centroid movement as calculated from manually segmented animals for animal in 4A (n = 25). (PDF) [file pcbi.1009942.s006.pdf]

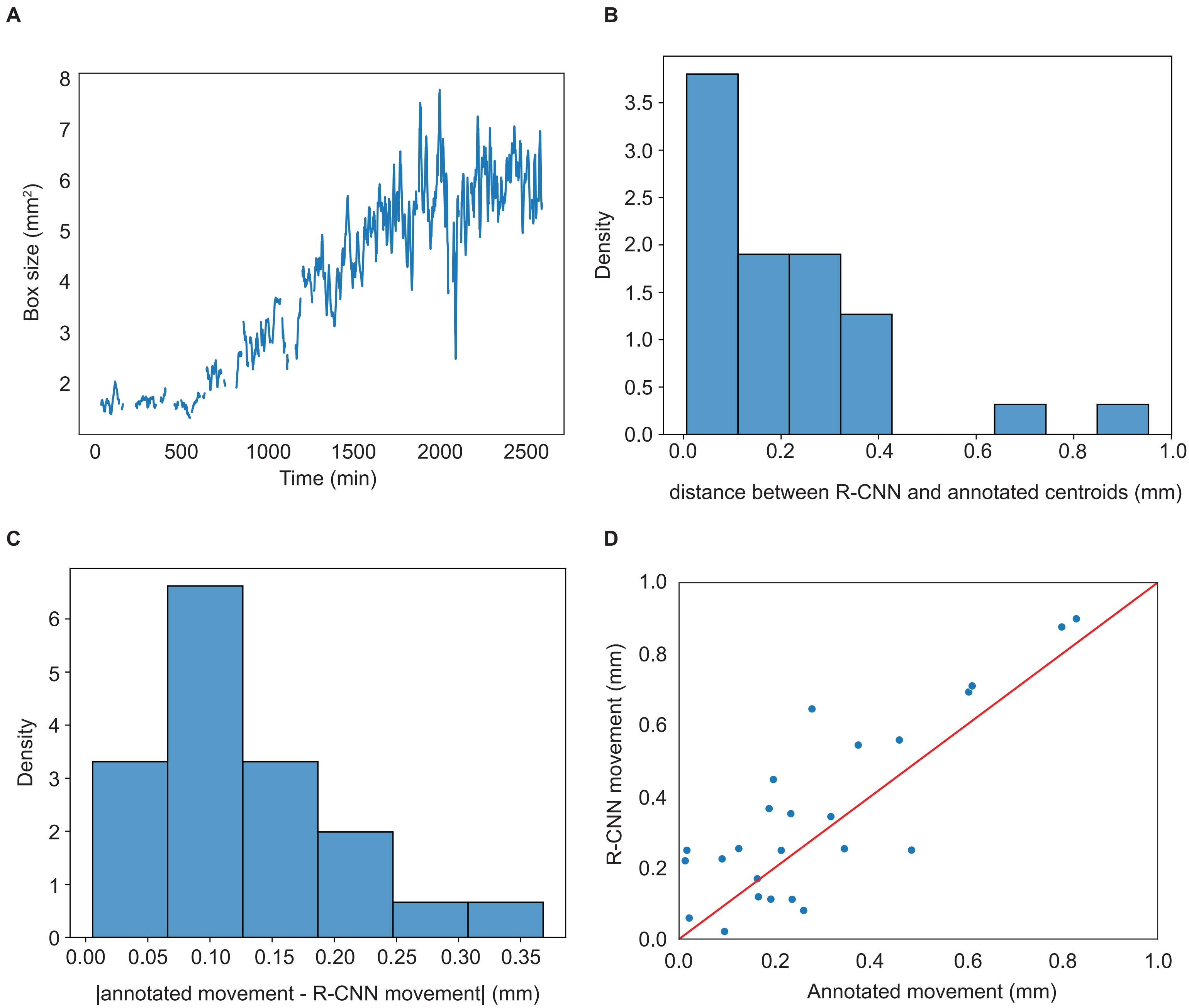

Supplemental Figure 6. Accuracy of tracking in development

A. Change of bounding box size over time using WoP Faster R-CNN for animal depicted in 4A. Smoothed using a moving window average over 10 time points (10 minutes). Where no animal is detected, line is not connected.

B. Density histogram of distances between box centroid as detected by WoP Faster R-CNN model and centroid of annotated worm shape for animal in 4A (n=30 time points).

C. Density histogram of difference between movement calculated from annotations and movement calculated from WoP Faster R-CNN model for animal in 4A (n=25 time points).

D. Centroid movement as calculated by the WoP Faster R-CNN model vs. centroid movement as calculated from manually segmented animals for animal in 4A (n=25)
