# Supplementary material for: Deep learning for robust and flexible tracking in behavioral studies for C. elegans
Source: PLoS Comput Biol. 2022 Apr 8;18(4):e1009942. doi: 10.1371/journal.pcbi.1009942 (PMC9020731; doi:10.1371/journal.pcbi.1009942)
Supplement: S7 Fig — A. Linear vs. angular velocity probability plot, calculated as described in Stern et al. (2017) [13] with centroid data from Stern et al. (2017) [13] Black dashed line shows split used to classify roaming vs. dwelling states. B. Linear vs. angular velocity probability plot, calculating angular velocity by using the Stern et al. (2017) [13] centroids at the current time as well as the centroid one minute in the past and one minute into the future. Black dashed line shows split at 90 degrees/min angular velocity used to classify roaming/ dwelling states with 79% accuracy based on ground truth classification in (A). (PDF) [file pcbi.1009942.s007.pdf]

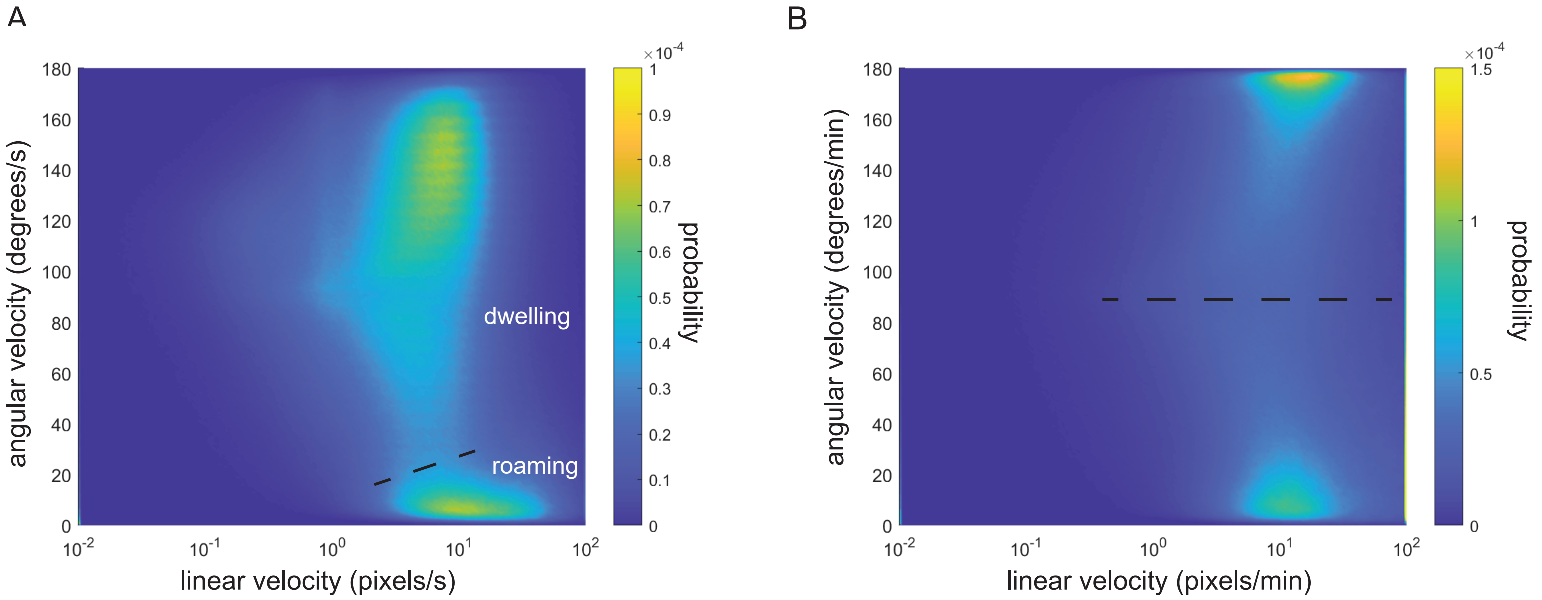


**Supplemental Figure 7. Accurate classification of roaming and dwelling at reduced sampling frequency**

A. Linear vs. angular velocity probability plot, calculated as described in Stern *et al*. with centroid data from Stern *et al*. Black dashed line shows split used to classify roaming vs. dwelling states.

B. Linear vs. angular velocity probability plot, calculating angular velocity by using the Stern et al. centroids at the current time as well as the centroid one minute in the past and one minute into the future. Black dashed line shows split at 90 degrees/min angular velocity used to classify roaming/ dwelling states with 79% accuracy based on ground truth classification in (A).
