# Supplementary material for: Deep learning for robust and flexible tracking in behavioral studies for C. elegans
Source: PLoS Comput Biol. 2022 Apr 8;18(4):e1009942. doi: 10.1371/journal.pcbi.1009942 (PMC9020731; doi:10.1371/journal.pcbi.1009942)
Supplement: S8 Fig — Histogram of IoU values for bounding boxes detected by the WiCh Faster R-CNN model compared to bounding boxes of hand annotated, segmented worms of the same frame. (n = 2550 frames). (PDF) [file pcbi.1009942.s008.pdf]

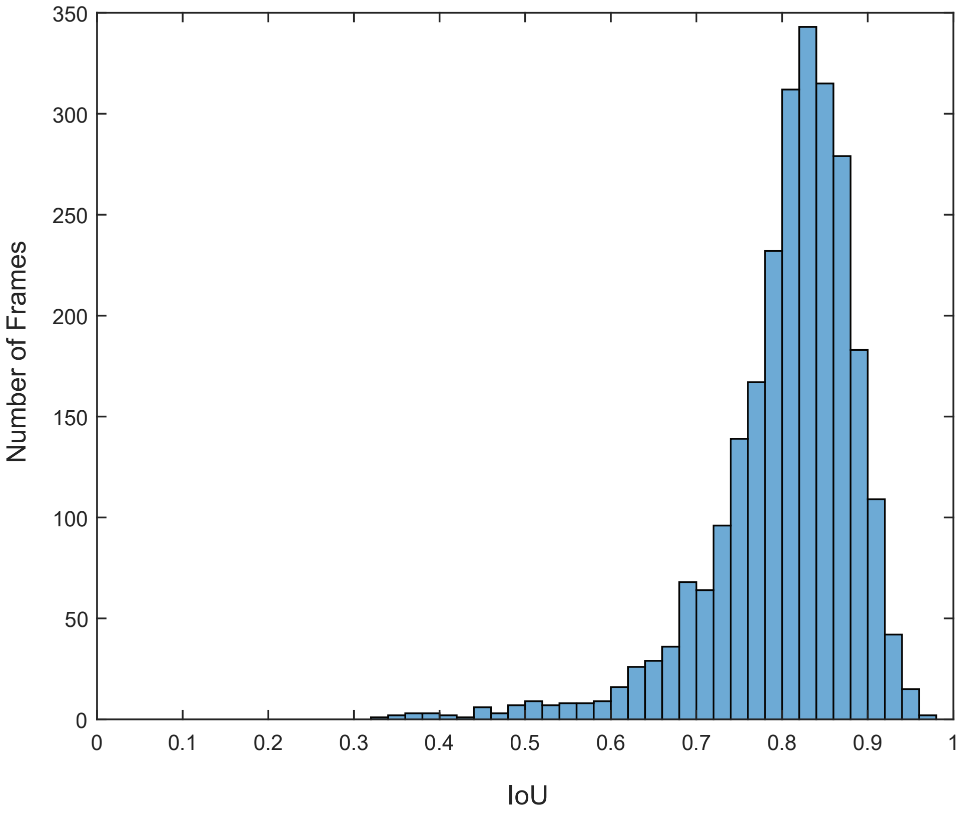

Supplemental Figure 8. Accurate detection of worms using the WiCh model

Histogram of IoU values for the bounding boxes detected by the WiCH Faster R-CNN model and the bounding box of the hand annotated, segmented worms of individuals shown in Figure 4B (n = 2550 frames)
