# Supplementary material for: Deep learning for robust and flexible tracking in behavioral studies for C. elegans
Source: PLoS Comput Biol. 2022 Apr 8;18(4):e1009942. doi: 10.1371/journal.pcbi.1009942 (PMC9020731; doi:10.1371/journal.pcbi.1009942)
Supplement: S9 Fig — A. Representative example frames of issues with segmentation using Ilastik within the same video. Even after training at least 50 frames (including a frame from the same video) the classification predictions and subsequent segmentations truncate the worm. Blue denotes background, yellow marks the worm, and red marks the egg objects. B. Representative example frames of issues with segmentation using Ilastik across similar videos. All frames were taken under the same imaging condition. (top) Prediction of pixel classification using the trained model. The model was trained with at least 50 images prior. Blue denotes background, yellow marks the worm, and red marks the egg objects. (bottom) Segmentation of objects based on the predictions. Note the truncation of worms and the misclassification of eggs as worms. (PDF) [file pcbi.1009942.s009.pdf]

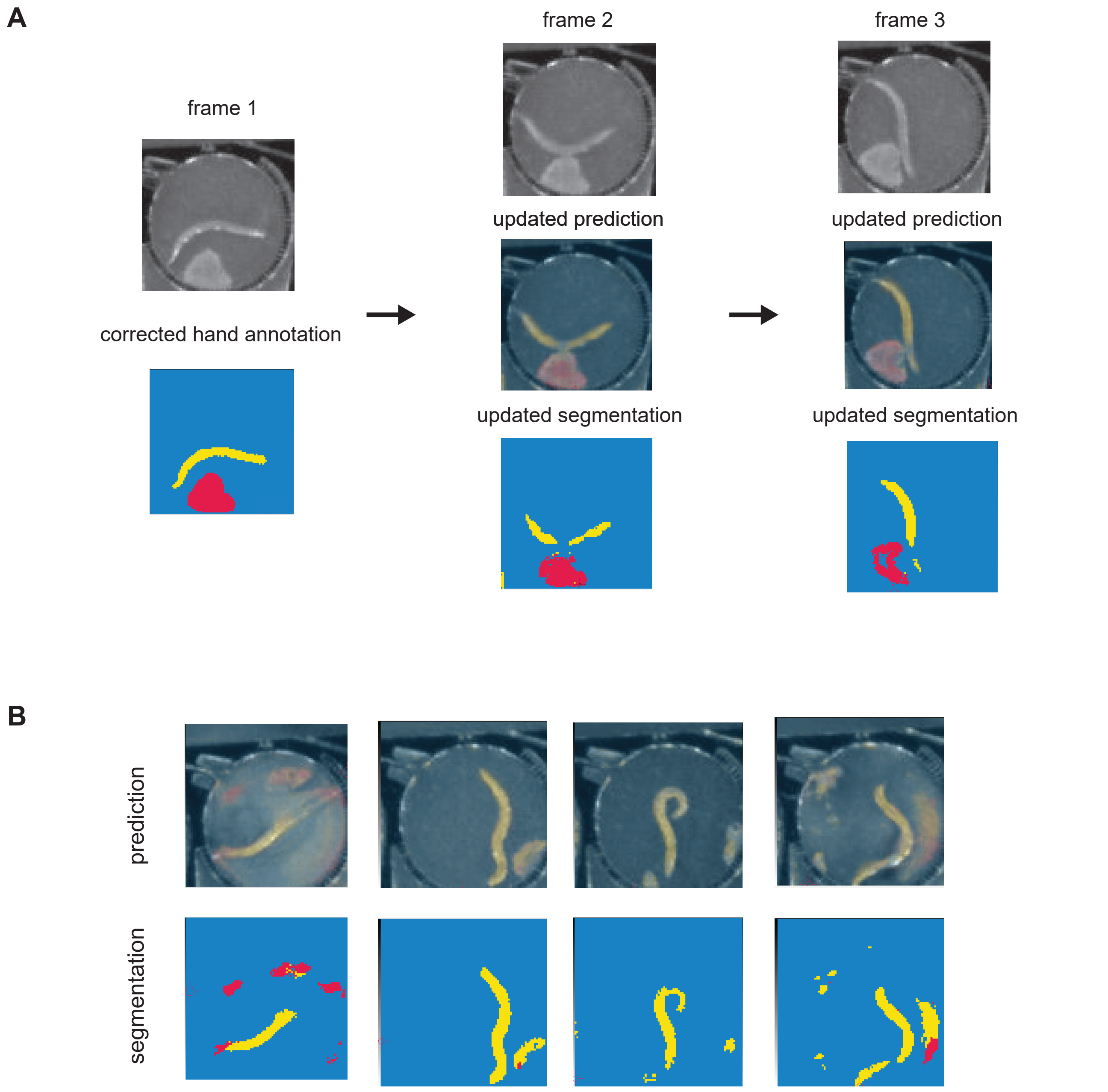

Supplemental Figure 9. Limitations with existing machine learning-based segmentation tools

A. Representative example frames of issues with segmenation using Ilastik within the same video. Even after training at least 50 frames (including a frame from the same video) the classification predictions and subsequent segmentations truncate the worm. Blue denotes background, yellow marks the worm, and red marks the egg objects.

B. Representative example frames of issues with segmentation using Ilastik across similar videos. All frames were taken under the same imaging condition. *(top)* Prediction of pixel classification using the trained model. The model was trained with at least 50 images prior. Blue denotes background, yellow marks the worm, and red marks the egg objects. *(bottom)* Segmentation of objects based on the predictions. Note the truncation of worms and the misclassification of eggs as worms.
