# Supplementary material for: Deep learning for robust and flexible tracking in behavioral studies for C. elegans
Source: PLoS Comput Biol. 2022 Apr 8;18(4):e1009942. doi: 10.1371/journal.pcbi.1009942 (PMC9020731; doi:10.1371/journal.pcbi.1009942)
Supplement: S1 Table — (DOCX) [file pcbi.1009942.s010.docx]

| Data set | Metric | Ground Truth | Faster R-CNN |
| --- | --- | --- | --- |
| Developmental (Fig 3) | Average velocity | 7.19 pixels/min | 9.49 pixels/min |
| Developmental (Fig 3) | Peak velocity | 30.61 pixels/min | 33.14 pixels/min |
| Stern *et al.*(2017) [1] | Average velocity | 3.78 pixels/s | 3.63 pixels/s |
| Stern *et al.* (2017) [1] | Peak velocity | 13.99 pixels/s | 9.69 pixels/s |

**S1 Table**. Peak and average velocity for developmental data set and data from Stern *et al.* (2017) [1]

**References**

1. Stern S, Kirst C, Bargmann CI. Neuromodulatory Control of Long-Term Behavioral Patterns and Individuality across Development. Cell [Internet]. 2017 Dec 14 [cited 2018 Jan 4];171(7):1649-1662.e10. Available from: http://www.ncbi.nlm.nih.gov/pubmed/29198526
