# Supplementary material for: Deep learning for robust and flexible tracking in behavioral studies for C. elegans
Source: PLoS Comput Biol. 2022 Apr 8;18(4):e1009942. doi: 10.1371/journal.pcbi.1009942 (PMC9020731; doi:10.1371/journal.pcbi.1009942)
Supplement: Attachment — Submitted filename: responses_frcnn_bates_le.pdf [file pcbi.1009942.s011.pdf]

We greatly appreciate the comments and suggestions from the reviewers and the editors. We have addressed all of the points in the revised manuscript. Below is a point-by-point response detailing changes we have made. The original comments are in Times New Roman in black and our responses are in Arial in blue.

1) Although the reviewers were very positive about the fact that an attempt had been made to make the tools available for general use without a high technical bar, in practice, the reviewer who tried to use the provided software was unable to get it working. It is suggested that you actively test the ability of a third party to use the software and make any modifications required in the tools or instructions to ensure the this is straightforward.

We appreciate the reviewers bringing this to our attention. We provide an updated version of the notebook (https://github.com/lu-lab/frcnn-all-in-one) along with a more robust saved file format for the trained models to ensure it works in the future. In addition to making it more robust for future use, we have also combined the annotation step (necessary for dataset preparation) with the Faster R-CNN training step into a single web-based notebook, which will be much more convenient for any software users.

2) Both reviews note that there is rather limited discussion of alternative approaches that use Deep Learning methods for animal tracking (such as Deep Lab Cut, LEAP). As mentioned in the reviews, sometimes these methods are being used for significantly different problems such as pose extraction vs. localization, but it would be helpful to make discussion of these differences more explicit. Nor is there reference to previous applications of the Faster R-CNN method to animal tracking, yet it has been used in several such applications to date, so providing more references and comparisons would be appropriate.

We appreciate the comment, and have added a broader discussion of other alternative deep-learning approaches and other applications of Faster R-CNN to animal tracking in the introduction (lines 88-90, 105-107). In addition, we provided greater clarity in the advantages of using the Faster R-CNN method as a localization method in place of more complex pose extraction or pixel classification techniques (lines 90-96, 545-551).

3) The reviewers note some situations in which the tracking seems less successful, and as they suggest, more discussion and analysis of the limitations of the method could be valuable.

We have added additional discussion of the circumstances where tracking is less successful. This includes emphasizing the egg detection model as a tool suited for trend-spotting in large datasets rather than highly accurate quantification, which we expand upon in lines 286-287, 292-293, and 297-300. We also discuss the potential for mutants (lines 367-369, 543-545) or smaller/ younger animals (lines 530-533) to effect successful tracking. We further emphasize that while the Faster R-CNN method we present is more generalizable than other methods, there are still limitations to that generalizability if the training data are too limited, for example in Figure 1B as brought up by Reviewer 1 (see lines 224-229 and lines 541-545).

Reviewer's Responses to Questions

Comments to the Authors:
Please note here if the review is uploaded as an attachment.

Reviewer #1: In this paper, Bates et al. describe a deep learning pipeline for identifying the locations of C. elegans L2-Adult and eggs in images. They show convincingly that it does an excellent job for this task despite variation in lighting conditions, decoys like worm tracks in the image background, and worm size. They illustrate its use on agar plates and in a microfluidic device, and for different purposes - fecundity assays, tracking movement, and growth measurements. They also have made the software available in an extremely easy to use form via well documented online jupyter notebooks off their github site.

First just a comment about software availability. Their web-based implementation of the software is just excellent, and it would be great if this became the standard expectation for this type of methods paper. All too often algorithms are described without any available implementation or there is an implementation but it is difficult to install or get to work or depends on extra software. By using online jupyter notebooks and open tools like colab, Bates et al. avoid these problems and make their software simple to use and access.

The paper is straightforwardly written and the results are well described. There are some things that could use some more explanation, however.

1) Lines 520-525 are really important! But they come at the very end. The method is framed as being able to "identify and detect worms" (eg. line 98). And of course it does that in a way. But it seems like it is more accurate to say that it identifies the _locations_ of worms. It identifies a bounding box that contains where a worm is but doesn't actually identify the worm's specific pixels. This is an important distinction for two reasons: (a) It means that this method is good for certain types of questions (line 518) but not others, and that it would need to be combined with something like Mask R-CNN (line 523) to give it that capability; (b) a lot of alternative methods that one might use (Supp fig 9) are actually focused on identifying the worm's pixels and so it's not really an apples-to-apples comparison. However, for any given application the comparison may be perfectly valid because if a bounding box is sufficient to answer the question then other methods are doing a worse job just to try to get extra information that's not needed. It's a good insight to realize that the location (and not the worm shape) is all you need to answer specific questions and that taking this distinction seriously makes it possible to develop methods like their WoP and WiCh that do this very well. But I didn't get this distinction until the very end after I'd spent the paper wondering why they were only looking at bounding boxes and not actually identifying worm pixels. I'd suggest adding a paragraph earlier making this point and also going into more detail about just why "the annotation necessary to train such a [segmentation] model is significantly more intensive." (line 525).

We have added clarification that the output of the Faster R-CNN architecture is bounding boxes in our introduction as well as more discussion of the potential benefits and trade-offs between pixel-wise classification methods and object detection methods like Faster R-CNN

(lines 88-96). We also elaborate on the additional labor involved in creating a training set for a deep learning model that can segment instances of objects (lines 571-574).

2) Because it identifies bounding boxes of worms, the orientation of the worms seems important. If a worm is oriented along the X or Y axis, then the bounding box will be thin and long. If it is oriented 45 degrees from that, then the bounding box will be square and have maximum area. This arbitrary orientation dependence seems like it would have an impact not just on where the centroid is but also on any overlap or area based metrics like loU in figure 4. As a suggestion, why not optimize the bounding box by rotating the image, identifying bounding boxes at each angle, and then picking the one with the smallest area (or some other metric like the intersection of all the boxes)? My speculation is that optimizing the orientation of the box might reduce some of the variability in some of the data (e.g. Fig S6A). Perhaps the intersection of all (or most) of the boxes might also be able to trim off pixels outside the convex hull of the worm. The downside would be that it would take longer to go through different angles, but there could be a way to do this in a way to cut down the number of angles tested - like a logarithmic search with the standard orientation and 45 degree rotation as starting points.

We thank the reviewer for the interesting suggestion. We agree that there are likely many additional ways that the bounding box information could be used. Here, we show several straightforward ways that bounding box information could be used to infer useful metrics from large datasets with an eye to fast but accurate localization of animals. While we believe it to be outside the scope of this paper to develop more advanced localization procedures and behavioral metrics such as those the reviewer suggests, we agree with the reviewer that it is a very interesting direction for future work.

We also agree that the centroid of the bounding box is not necessarily the centroid of the worm (for example, see the comparison between the bounding box centroids and the center of mass of pixel-wise hand-segmented worms in Figure 3A & B). We found that in practice, the difference between the two sets of centroids was on the order of just a few pixels for images on the scale of thousands of pixels in each dimension (see lines 389-395). Likewise, our IoU metric may be impacted by the orientation of the worm in individual frames, but we expect it to still provide a reasonable estimate of the worm's movement, especially when observing on timescales much longer than that of the measured behavior (see lines 486-488, 491-495). As the reviewer suggested here, there is a trade-off between accuracy and simplicity, and we took the side of computational simplicity and expediency for a small cost in accuracy.

3) The paper doesn't deal with L1s or early L2s at all. Is this a limitation of their microscopy setup where the magnification was too low to get a lot of pixels for these worms? Or is it that because of background or whatever the software just doesn't work well for these worms and more magnification wouldn't help?

This is purely a microscopy limitation of the system used to collect this long-term data. We have seen with other datasets collected on other microscopy systems that L1 and L2s can

also be detected. We have indicated this is a hardware limitation in our manuscript (lines 530-533).

4) In figure 1B, the system doesn't seem to work well for a few of the worms towards the bottom which don't seem unusual conformation-wise (the ones in the middle, not the ones on the lower right which are clumped). What is going on there? What is it about these worms that makes them undetected? Along those lines, in the upper right there is a worm doing a turn that is not identified. Is this just a shortcoming of not enough turning worms in the training set or does the model have difficulty with worms of this shape?

We appreciate the reviewer's attention to this point. It is likely that the worm turning in the upper right was not identified because, as the reviewer suggests, only a very small percentage of training images contained animals that were mid-turn. The training images were collected from animals behaving on solid agar media, where animals only occasionally take on postures with very deep bends, while in lower-resistance liquid media this type of posture is more common. Several of the animals in the middle-bottom of Figure 1B that were not identified also contain deeper bends that are rare in the training set. The remainder of the unidentified worms in the middle-bottom of Figure 1B were likely screened out by the confidence criteria we apply to ensure low false positive detection of worms. Faster R-CNN also provides a confidence level that we use to screen out objects (in this case worms) with a confidence level less than 80%. We used the same confidence criteria for the animals in Figure 1B as for the animals in Figure 1A to decide whether to consider detections as actual worms. Considering that the contrast levels between worms and background is very different in these two sets of images, and that the Faster R-CNN model used for detection of animals in Figure 1B was only trained with images like those in Figure 1A, it is not surprising that this level of confidence may need to be adjusted. We have added a discussion of this in our manuscript (lines 224-229).

5) The model seems like it does decently for identifying eggs in complex environments, but it is hard to tell where on the spectrum of typical plates their data fell. There are definitely worm tracks in the images in Figure 2A, but is this a typical background, and easy background, more worm tracks and intensity furrows that usual? It's hard to evaluate whether the numbers in lines 222 & 223 would be typical or not. However, the authors do appropriately make the comment that this egg detection is probably well suited for identifying trends (rather than precise counts) and so it's probably the case that identifying trends would be decently robust to image background variation.

The animal tracks visible in Figure 2A are within the typical range of bacterial density for animal behavior experiments. Many behavioral assays have even lower bacterial density than what we use here in order to promote food searching behaviors that produce faster and more consistent movement. We have added a more thorough discussion of the limitations of the egg detection method to address this comment, as well as Reviewer #2's third comment (see lines 291-292, and below for response to Reviewer #2's comment).

Overall, this is a nice method, described in a well-written paper, provided in an excellent web-based format, and will be quite useful for a lot of worm assays. It makes an important implicit point (which should become explicit) that some kinds of information are easier to extract from images than others and that if this easier data is the essential data, you'd do better to focus on it and not try to do everything.

Minor points: Line 435: left -> top

We have corrected this error.

Some of the methods have been described in previous papers (e.g. the microfluidic device, palmitic acid). Some are standard worm methods (culture on agar plates). The Raspberry Pi imaging setup is not cited, but neither is it described completely enough for someone to be able to replicate it. There is a mi-pi repository on the Lu lab github page. If this is the system used, I'd suggest adding a link around line 556.

The mi-pi repository is the core software used as part of the imaging setup used in Figure 3. We have updated our manuscript to include the link to the Github page (see lines 611-612).

Reviewer #2: While classic image processing techniques have enabled us to extract rich datasets that accurately describe the behavioural repertoire of C. elegans, there are still many behaviours that cannot be analysed using this approach. This leads to time consuming manual analysis and precludes larger scale studies. Bates et al., have utilised a deep learning approach to overcome this challenge. Using Faster R-CNN they trained and validated 3 models that have been tuned to look at 3 behaviours that are notoriously challenging to analyse in an automated way; development, egg laying, and aging. They have also established a web platform that allows other researchers to apply these models to their own data and train their own models. Their approach is also flexible and performs well under different environmental conditions, another important consideration for researchers who can't afford to set up a new imaging system. This work will be incredibly beneficial to many researchers who work with C. elegans.

I recommend the publication of this manuscript in PLOS Computational Biology; however, the authors need to first address the following comments.

1. I followed the 'data annotation' notebook until the 'annotating images' section and when I ran the cell I could see 'loading widget…' but no GUI emerged after several minutes. I didn't test the training notebook as I got stuck at this point in the annotation notebook. I used the 'Faster_R_CNN_inferencing' notebook to test the egg model on data from our lab. I ran all the code and didn't encounter any errors; however, no worms or eggs were detected. I then tested a video from the OpenWorm database to check if my data was the problem and again no worms were detected. I tried inputting the data as videos and as a folder of images and no worms were

detected. I lowered both 'target_min_scores' values to 0.1 and still no worms were detected. I wondered if this was just an issue with the egg model, so I also tested the development model, and again no worms were detected. I may be doing something wrong or there is an invisible bug that's preventing the model from being utilised correctly. I hope the authors can resolve this issue or provide additional instructions.

We appreciate that the reviewer took the time to test our web-based tools. We have updated our Faster R-CNN training notebook so that it includes a widget for annotating images and have re-tested the notebook to ensure it works. We believe having annotation within the training notebook will also be much more convenient for users.

We have also resolved the reported issues regarding the training and inferencing notebook. We provide an updated version of the notebook, along with more robust saved file formats for the trained models used in the paper, found on our FigShare.

2. I think the authors should give a broader overview of deep learning approaches used for behavioural analysis in other species in the introduction. For example, DeepLabCut (http://www.mackenziemathislab.org/deeplabcut). Why did the authors choose Faster R-CNN over DeepLabCut?

We thank the reviewer for their suggestion and incorporated a broader discussion on deep learning approaches for behavioral analysis in our manuscript (lines 88-90, 105-107). In addition, we clarified the advantages of using Faster R-CNN and object detection over existing techniques which output estimated poses (including computational cost, ease of training, etc.) in the text (lines 90-96, 528-540).

3. Supplemental movie 1 shows the performance of the egg model on a video from the OpenWorm database. It appears the eggs are only counted after the worm has moved away from them and not immediately as they are laid (egg laying event). In many cases C. elegans researchers want to measure the time an egg is laid so that the interval between egg laying events can be analysed. I think the authors need to be more explicit about the limitations of their egg detection model for measuring an egg laying event.

As the reviewer suggests, we have added language to point this out to readers and reiterate in the text that the egg-finding model is likely best used to identify *trends* in egg laying events (lines 285-286, lines 296-299).

4. Were all models trained on images from N2 worms? I'm wondering if the models will perform as well on worms with locomotion defects. Do the models take movement into consideration?

We trained our Faster R-CNN model exclusively on N2 images; however, the model does not take movement into account, and uses just the information from a single image to find objects. Thus, we do not expect that locomotion defects should have any effect on model performance. The mutant *tph-1*, which we used in Figure 2 with our egg-finding model, has a

locomotion defect, but it did not affect the detection of *tph-1* animals. We have clarified these points in the text (see lines 214-215 and lines 367-369).

5. I really appreciate that the authors took the time to provide clear instructions and resources so that other scientists could train their own models. How do you suggest other scientists validate their models? I think the authors should discuss the limitations of deep learning approaches being used more broadly by non-experts without the correct validation.

We agree with the reviewer about the importance of model validation, particularly when using black-box like analysis techniques. To enable researchers to validate their own trained models, we incorporated an additional validation step and further documentation in the Faster R-CNN training notebook (also see lines 547-549 in the manuscript). This will allow users to characterize their own trained models by extracting the AP values, similar to our own characterization in the manuscript (shown in Table 1).

6. The authors demonstrated how machine-learning image classification tools like Ilastik were not effective at segmenting the worms under different environmental conditions. Can the authors suggest any other deep learning segmentation approaches that might fair better? Or do they think deep learning is not the right approach for segmentation of worms?

In principle, other deep learning methods, such as Mask R-CNN, should work for segmentation. However, these techniques are difficult to use particularly for naïve users. They are often difficult to locally install or implement, require large training sets of data that are difficult to annotate, and need substantial computational power to train and perform inferencing across frames. In contrast, our paper provides an alternative approach. Instead of segmenting the individual to obtain posture, we perform object detection, examining the location of the individual in the form of bounding boxes. This in turn allows us to extract useful, commonly used behavioral information through less computationally complex methods.

7. Line 233 and 173: typo ‘lain’

Thank you – we have corrected both to ‘laid’.
